# Assessing the Effect of Marine Reserves on Household Food Security in Kenyan Coral Reef Fishing Communities

**DOI:** 10.1371/journal.pone.0113614

**Published:** 2014-11-25

**Authors:** Emily S. Darling

**Affiliations:** 1 Earth to Ocean Research Group, Simon Fraser University, Burnaby, BC, Canada; 2 Wildlife Conservation Society, Mombasa, Kenya; University of Western Australia, Australia

## Abstract

Measuring the success or failure of natural resource management is a key challenge to evaluate the impact of conservation for ecological, economic and social outcomes. Marine reserves are a popular tool for managing coastal ecosystems and resources yet surprisingly few studies have quantified the social-economic impacts of marine reserves on food security despite the critical importance of this outcome for fisheries management in developing countries. Here, I conducted semi-structured household surveys with 113 women heads-of-households to investigate the influence of two old, well-enforced, no-take marine reserves on food security in four coastal fishing communities in Kenya, East Africa. Multi-model information-theoretic inference and matching methods found that marine reserves did not influence household food security, as measured by protein consumption, diet diversity and food coping strategies. Instead, food security was strongly influenced by fishing livelihoods and household wealth: fishing families and wealthier households were more food secure than non-fishing and poorer households. These findings highlight the importance of complex social and economic landscapes of livelihoods, urbanization, power and gender dynamics that can drive the outcomes of marine conservation and management.

## Introduction

Coral reef fisheries support the livelihoods and food security of hundreds of millions of people in developing countries [1], [2], [3]. However, many coral reef fisheries are unsustainably exploited [4], [5] and coral reefs themselves are threatened by a variety of anthropogenic impacts [6], [7] leading to widespread concern that coral reefs will no longer provide adequate food security in the coming decades [8], [9]. Enhancing and maintaining food security is a critical goal for coastal communities in developing economies, even more so given the increasing threats to fisheries from climate change [6], [10]. An essential question is how to sustainably manage coastal and marine environments to improve coral reef biodiversity and food security [11], [12], [13].

No-take marine reserves are a popular tool to manage coral reef fisheries for ecological, economic and social benefits, including food security [11], [14]. The recovery of fish biomass inside marine reserves [15], [16] can spillover into nearby fished areas to increase fisheries yields [17], [18], [19]. Increased yields can provide economic benefits to fishers by increasing their total catch and income [14], [20] and it is often assumed that these direct fishery benefits will have indirect social and economic consequences for local communities in terms of increased food security and human welfare [10], [21], [22], [23]. However, these assumed links between biodiversity conservation and food security are often tenuous and narrow perspective that fails to take into account local social, economic and cultural realities [13]. For example, local marine management may not always have positive effects on food security. No-take marine reserves can impose costs on fishing livelihoods by reducing income and displacing fishing effort to overexploit adjacent areas [11], [24]. Additionally, rights and access to any management benefits may vary among social, ethnic or political groups within communities [13].

Understanding the effects of conservation and management actions for human wellbeing, such as food security, is critical for sustainable management [25] and it is surprising this remains poorly understood. While other studies have investigated the impacts of marine reserves on human nutrition and health [21], [26], household income and economic vulnerability [20], [27], few studies have quantitatively investigated the influence of marine reserves on household food security, defined as the ability of families to access safe, sufficient and nutritious food [28], [29]. Marine reserves and better managed fisheries can contribute directly to food security as a source of animal protein, essential fatty acids and micronutrients, and indirectly by providing livelihoods and income that can be used to purchase food [13].

Here, I assess the effect of marine reserves on household food security, measured by the consumption of animal protein, the diversity of major food groups in the diet, and how households cope with short-term food shortages. I evaluated the influence of two 40-year-old, well-enforced, no-take Marine National Parks on households near and far from each reserve in four coastal communities in Kenya. Specifically, I assessed the social and economic drivers of household food security, quantified whether the presence of a marine reserve affected household food security, and evaluated the contribution of fishing livelihoods and other socio-economic conditions to household food security.

## Methods

### Ethics statement

This study was approved by the Office of Research Ethics (#2010s0059) at Simon Fraser University. The Kenyan Office of Science and Technology provided research clearance (NCST/5/002/R/729) and I received additional permission to conduct interviews from the Office of the President and village leaders. All respondents provided verbal informed consent to participate in this study, which was documented on survey questionnaires. Written consent was not obtained because a verbal questionnaire was used; this consent procedure was approved by the SFU Office of Research Ethics board.

### Kenyan coral reef fisheries

The Kenyan coastline stretches 600 km from Somalia to Tanzania. Marine fisheries have been estimated to employ 20,000 people [30] and provide monetary income to ∼70% of coastal communities [31]. The coral reef fishery in Kenya is typical of small-scale, artisanal fisheries in developing countries around the world. Fishing is primarily a subsistence livelihood where fishers use low-technology gears (e.g., hand lines, nets, traps and spear guns) that target multiple species for a marginal catch of several kilograms per day [20], [32], [33]. The landed catch is then typically sold to local fish traders who will transport and sell the fish to nearby fish markets [34]. Fish that is not sold is then brought back to the household for consumption – many coastal households rely on fishing and other marine resources for their protein intake and livelihoods [30]. Thus, the Kenyan coral reef fishery supports a subsistence livelihood that provides food on the table and a cash income that can be used to purchase other food and essential items that contribute to food security.

Increasing fisheries exploitation and observed declines of sharks, turtles and reef fish led to the establishment of a series of Marine Protected Areas (MPAs) along the Kenyan coast beginning in 1968 [35]. Within each MPA, there is typically a small (<11 km^2^) no-take marine reserve (called a Marine National Park) that is effectively enforced against fishing and poaching by the Kenya Wildlife Service. These are some of the most effective no-take closures in the western Indian Ocean and have well-documented ecological and economic benefits, such as increased reef fish biomass [36], higher coral cover and diversity [33], [37], higher revenues and income for fishermen [20]. Fishers near marine reserves also typically have greater awareness of the impacts of pollution and market demands on marine resources than fishers far away from reserves, although this may also be associated with urbanization [38]. However, the effects of marine reserves or fishing livelihoods on household food security remain poorly understood.

### Household surveys

To assess the effects of marine reserves, fishing livelihoods and socio-economic variables on household food security, I conducted semi-structured interviews in four coastal fishing villages in Kenya during April 2010. Two villages were located less than 5 km from a no-take marine reserve with fishing grounds that were adjacent to the protected area: Anzuwani village adjacent to Kisite Marine National Park (MNP), and Uyombo village adjacent to the Watamu MNP (see map of study sites in Figure S1 in File S1). At the time of this study in 2010, both the Kisite and Watamu MNPs had been protected for nearly 40 years, since 1973 and 1972 respectively [35], [39]. In Anzwani and Uyombo villages, the marine reserve boundary was visible from the fishery landing site and fishers actively ‘fished the line’ of the reserve boundary. I also conducted interviews at two villages located more than 20 km away from a marine reserve. On the south coast, Kirudi village in Tiwi was a paired control for Anzuwani village (Kisite MNP), and on the north coast Msumarini village was a paired control for Uyombo village (Watamu MNP). Like many protected areas e.g., [50], [51], the two Marine National Parks chosen for this study were located in more remote locations >80 km away from the nearest major city and Kenya's second largest city, Mombasa (population: ∼1.2 million); I chose not to include the more recently protected (1991) and more urban Mombasa Marine National Park in this analysis (Figure S1 in File S1). While the city of Mombasa potentially introduces urbanization as a confounding factor, comparisons of fishing and non-fishing households and matching analyses on socio-economic attributes (see below) serve to tease apart the influence of urbanization [38]. The two ‘far from MPA’ villages, Msumarini and Kirudi, are >20 km away from the Mombasa MNP and >50 km away from their paired marine reserves. It is unlikely that Msumarini and Kirudi would be influenced by fisheries spillover from the Mombasa MNP; spillover from marine reserves, if observed at all, typically occurs on the order of hundreds of meters to several kilometers [17], [40].

I surveyed households using a systematic sampling design where every *i*th house (e.g., 2^nd^, 3^rd^, 4^th^) was selected, with *i* increasing with village size, to attain a target sample size of ∼30 households per village. Only households with a female head-of-household were considered; if no female head-of-household was present, we moved on to the next household. Households defined as a group of people living together and eating the same meals. During each interview, female heads-of-household were asked about their age, where they were born, years of formal education, the number, gender and age of people in their household, the number and type of occupations held by members of the household, estimated fortnightly expenditures and wealth (Table 1; see Table S1 in File S1). Household wealth was evaluated by a multivariate Material Style of Life index based on the presence or absence of various household possessions, such as a radio, cell phone, bicycle, toilet, electricity, type of cooking fuel and the building materials of the house [22], [38]. These items were combined in a Principal Component Analysis (PCA) where the first PCA axis explained 35.3% of the variation in wealth among households (see Figure S2 in File S1). This wealth axis described poorer households as having homes with dirt walls, dirt floors, and thatch roofs, and wealthier households as having homes with cement walls, cement floors, metal roofs and access to electricity and a latrine toilet. I used this composite score as an indicator of household wealth for subsequent analyses.

**Table 1:** Description and summary of social, economic, and food security variables surveyed in four coastal fishing villages in Kenya.

Indicator	Description	Range (min to max)	Mean (SD)
Age	Age of female head-of-household respondent	18 to 80	39.65 (14)
Education	Number of years of education by the female head-of-household	0 to 12	2.8 (3.62)
Household size	Number of people living in household	1 to 28	6.81 (3.84)
Household structure	Number of adults	1 to 10	2.32 (1.37)
	Number of children	0 to 18	4.65 (3.04)
Occupations	Number of total occupations (part-time or full-time) in the household and number of different jobs (occupational diversity)	0 to 6	2.39 (1.06)
Fortnightly expenditures	Cash expenses of the household standardized over a two week period (recorded in Kenya shillings)	350 to 15821	4665.15 (2656.62)
Wealth	Material Style of Life principal component axis from presence of absence of household possessions (radio, cell phone, bicycle, toilet, electricity, type of cooking fuel and house construction)	−1.89 to 6.14	0 (2.22)
Food security	No. of days per week that protein was consumed by household	0 to 7	4.42 (2.37)
	Diet diversity (number of seven major food groups consumed by the household over the past three days)	2 to 7	4.68 (1.34)
	Food Coping Strategies Index (FCSI) (frequency and severity of coping behaviours during the most recent dry season and monsoon season).	0 to 47.75	16.34 (10.62)

### Food security

I also asked respondents about the food security of their household. Food security is defined as the physical, social and economic access of people to safe, sufficient and nutritious food and can be a notoriously elusive concept to measure [28], [29]. I attempted to quantify food security in several ways to encompass both consumption trends and responses to changes in food availability [28]. Consumption was assessed through a three-day diet recall of foods that the respondent had prepared for the household. A three-day recall was chosen because it was an appropriate time scale that the respondent could accurately remember and also one that captured most of the household's food repertoire [41], [42]. From each diet recall, I calculated the estimated weekly frequency of animal protein consumption (fish, meat, poultry, eggs) and a 7-point score of diet diversity. For diet diversity, each food item prepared by the household was classified into 7 major food groups based on [41]: *1*) starchy staples of grain, roots or tubers; *2*) legumes; *3*) dairy; *4*) meat, poultry, fish, or eggs; *5*) vitamin A-rich fruits and vegetables, such as pumpkin, squash, carrots, green leafy vegetables, mango, papaya; *6*) other fruits and vegetables; and *7*) foods made with oil, fat, or butter. The diet diversity score was a number from 0 (no food groups present in diet during the 3-day diet recall) to 7 (all food groups present in diet). All interviews were conducted over a three-week period in the dry season (April) to minimize potential differences in diet between the dry and rainy seasons that can be typical of coastal Kenyan villages [43].

I also measured a food coping strategies index (FCSI) to characterize the frequency and severity of the coping strategies used by a household to deal with short-term food insecurity [29]. A household may also employ longer-term strategies to cope with food shortages, such as migration back to a family homestead or to an urban area where work is more readily available; however, this was beyond the focus of the current study. For short-term coping strategies, six general behaviours have been identified for rural households in sub-Saharan Africa: 1) eating less preferred foods; 2) limiting portion sizes; 3) borrowing food or money to buy food; 4) preparing food only for the children as a type of ‘maternal buffering’; 5) skipping meals; and 6) going without food for whole days [29]. For each coping behaviour, I asked respondents to estimate the average number of times per week during the dry and rainy seasons (see below) that they employed that behaviour: never (0 days per week), occasionally (1–2 days per week), often (3–6 days per week), and always (7 days per week). I also asked each respondent about their perception of how worried they would be to adopt each coping strategy, which was used to compare with previous studies of sub-Saharan households [29]. Perceptions were scored on a three-point severity scale from “not worried (score of 1), “a little worried” (score 2), to “very worried” (score 3). There was generally good agreement between the respondents' perceived severity scores and weightings previously developed for each coping strategy for sub-Saharan African households (see Table S2 in File S1). Previously published severity scores of [29] were used for all analyses. To calculate the coping strategies index, I multiplied the weekly frequency of each coping mechanism by its severity score and summed these values across the six coping strategies to obtain an FCSI for each household, following the methods of [29].

Respondents were also asked about food coping strategies in two seasons: the dry *kaskasi* season (when all interviews occurred) and the most recent wet *kusi* season. These two seasons were initially kept separate because the dry season is typically better for fishing livelihoods while the wet season is better for agricultural livelihoods. I hypothesized that food insecurity would be higher for fishing households in the wet season when the sea is often too rough to fish, and that non-fishing households would experience greater food insecurity in the dry season when it is too harsh for many crops to grow. However, this hypothesis was not supported by the interview responses. When asked, “What season is better to have enough food for your family?”, there was no evidence for seasonality between fishing (dry season better = 30 households, wet season = 19, no difference = 6) and non-fishing households (dry season = 22 households, wet season = 25, no difference = 11; Fisher test, *p* = 0.18). There was also no significant difference in the food coping strategies index by season (two-way ANOVA, *F* = 0.003, *p* = 0.95) or an interaction between season×livelihoods (*F* = 0.95, *p* = 0.33). I thus calculated an annual food coping strategy index as the average of these two seasons that was used in all FCSI analyses.

### Data analysis

Non-parametric Wilcoxon rank sum tests were used to evaluate differences in baseline socio-economic characteristics between households with fishing and non-fishing livelihoods, and between households near and far from marine reserves. All tests were corrected for multiple comparisons using the False Discovery Rate (Pike 2011). General linear models with model selection in an information-theoretic framework were used to quantify the drivers of household food security. For each of the three food security metrics (frequency of weekly protein intake, diet diversity score, and FCSI), I evaluated the relative support for models with respondent age, education, household size (the total number of adults and children), the number of household occupations, fortnightly expenditures, household wealth (PCA axis 1 score, Figure S1 in File S1), the primary occupation of the household (fishing/non-fishing), marine reserve distance (near/far) and the interaction between fishing and marine reserve distance (Table 1). I included a fishing×marine reserve interaction because I hypothesized that proximity to marine reserves would have more positive effects on fishing households than non-fishing households if marine reserves have more direct economic benefits for fishers more than non-fishers. Each predictor was an independent variable in the analysis as indicated by variance inflation factors (VIF<2 fro all predictors; [44]). I also centered and standardized each predictor prior to analysis, which allowed for direct comparisons of the relative contribution of each predictor's effect size [45].

Using a multi-model information-theoretic approach, I competed models with all possible combinations of the predictor variables and compared them using Akaike's Information Criteria corrected for small sample size (AICc). For each predictor, I calculated the average effect sizes and 95% confidence intervals for models within top model set (as determined by the 95% confidence model set; [45]). Model diagnostics were performed to check for homogeneity and normality of the residuals of the global model. Two fishing households in Uyombo village had large multi-family households (23 and 28 people) and were removed as outliers from the analysis (following [44]). Model selection was completed using the package “MuMIn” [46] in R [47] following the protocol of [45].

To directly evaluate the influence of marine reserves on food security, I also used matching methods as a complementary approach to general linear models. Matching methods are commonly used with observational data in economics, epidemiology, medicine and political science to estimate causal effects (see review by [48]). Matching methods are also used for conservation impact evaluations of protected areas to address differences in social and economic baselines between control and treatment units. This *ex post* approach effectively balanced a diverse datasets of “apples” and “oranges” by selected a subset of “apple to apple” pairs that are used for all statistical comparisons [49]. For example, studies of protected areas and poverty reduction in Costa Rica and Thailand compares land parcels that have been matched for similar forest cover, land use, distance to major cities and baseline poverty to show protection can alleviate poverty when properly matched samples are compared [50], [51]. In this study, comparable pairs of control (far) and treatment (near marine reserve) households were matched based on household size, number of occupations, wealth and fortnightly expenditures. I selected pairs of households using a genetic matching optimization algorithm [52], [53] that significantly improved the similarity of covariate distributions as compared to the unmatched sample of households. Paired *t*-tests were then used to compare food security between the matched pairs of households. All matching analyses were performed with the package “Matching” [53] in R [47].

Finally, I used non-parametric multivariate analyses of variance (MANOVA) to compare the weekly consumption of the seven major food groups (i.e., household diets) between households near and far from marine reserves and between fishing and non- fishing livelihoods. Post-hoc differences in household diet were assessed using univariate Wilcoxon rank sum tests corrected for False Discovery Rate [54].

## Results

A total of 113 interviews were conducted with female heads-of-households in four villages; between 24 and 32 households were surveyed in each village (mean ± SD, 28.0±3.5 households; Table S1 in File S1). Respondents in fishing and non-fishing households had similar ages, years of education and fortnightly expenditures (Wilcoxon rank sum tests, all *p*>0.05). The majority of female respondents were originally from the Coast Province (100 out of 113, 88%) and 13 respondents were from elsewhere in Kenya or neighbouring Tanzania (12%). There was no different in respondent origin (i.e., Coast or non-Coast) across the four study sites (logistic general linear model, *P* = 0.10). While I did not specifically inquire about ethnicity or religious faith, each village was comprised of predominantly Swahili ethnicities with predominantly Islamic faith and a Christian minority.

In each village, I surveyed households where the primary livelihood was fishing (*n* = 55) and households where the primary livelihood was not fishing (*n* = 58, e.g., farming, construction or small business owners). Fishing and non-fishing households were comparable in some socio-economic aspects while different in others (Table 2). Fishing households typically had more people (size: *W* = 2098.0, *p* = 0.006), more household jobs (*W* = 1960.0, *p* = 0.014) and were poorer on average (wealth: *W* = 1066.5, *p* = 0.014) than non-fishing households (Table 2). Similarly, households near a marine reserve also differed from households far from a reserve in some social-economic characteristics but not others (Table 2). Near marine reserves, respondents had, on average, more years of education (*W* = 1184.5, *p* = 0.04), and households relied on more jobs (*W* = 1153.0, *p* = 0.04) and were poorer (*W* = 2200.0, *p*<0.001) than those further away from a marine reserve.

**Table 2:** Summary of socio-economic characteristics and food security metrics from fishing and non-fishing households, and households near vs. far from a no-take marine reserve.

		No. surveys^a^	Household size	No. adults	No. children	Age, years	Education, years	No. of jobs	Household wealth (PC1)	Fortnightly expenditures (KSh)	Protein consumption (days week^−1^)	Diet diversity (no. food groups)	Food Coping Strategies Index
**Household Livelihood**	Fishing	53	7.26 (2.56)	2.13 (0.59)	5.17 (2.39)	37.09 (11.26)	2.13 (3.12)	2.58 (1.03)	−0.73 (1.63)	4436.57 (1893.44)	5.42 (1.93)	4.49 (1.53)	17.15 (11.52)
	Non-fishing	58	5.74 (3.04)	2.24 (1.23)	3.78 (2.70)	41.72 (15.98)	3.43 (3.98)	2.10 (0.87)	0.71 (2.49)	4681.02 (3073.75)	3.50 (2.37)	4.88 (1.14)	14.69 (8.56)
**Location with respect to marine reserve**	Near, <5 km	52	6.65 (2.61)	2.17 (0.83)	4.77 (2.41)	36.77 (12.54)	3.62 (3.77)	2.56 (0.98)	−0.95 (1.49)	4526.75 (2583.18)	4.98 (2.32)	4.46 (1.42)	15.89 (10.12)
	Far, >50 km	59	6.31 (3.16)	2.20 (1.10)	4.15 (2.82)	41.93 (14.96)	2.10 (3.39)	2.14 (0.94)	0.88 (2.44)	4597.39 (2580.29)	3.92 (2.32)	4.90 (1.26)	15.84 (10.19)

a Number of households.

Mean values and standard deviations are shown for each variable.

**Figure 1:**
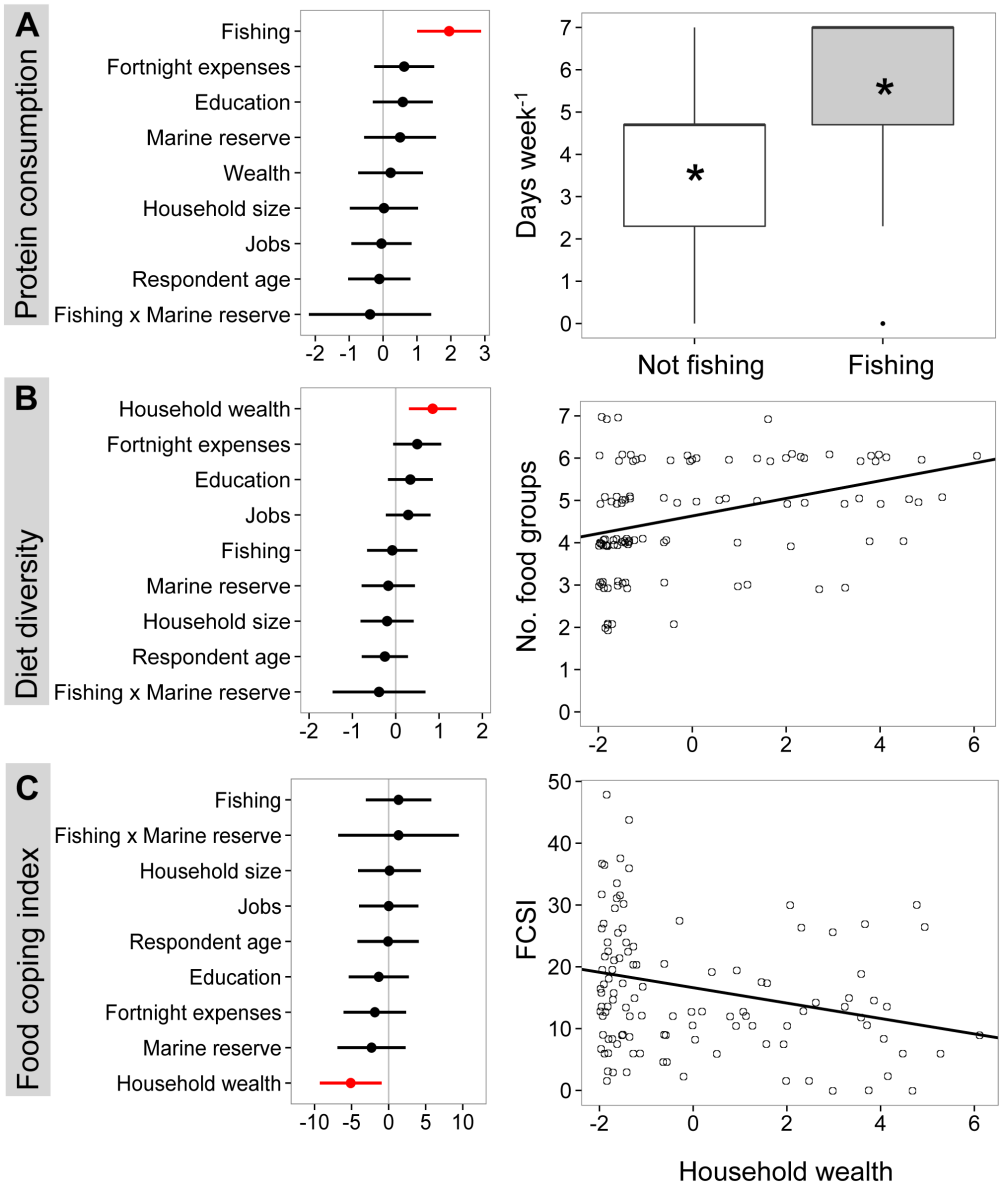
Importance of socio-economic characteristics and proximity to marine reserves for household food security in Kenyan coastal communities. Food security was described by three metrics: (A) protein consumption, (B) diet diversity and (C) food coping strategies. Panels on the left show averaged effect sizes and 95% confidence estimates from multi-model averaging; the line at zero indicates no effect. Significant predictors (where the 95% confidence interval does not overlap zero) are highlighted in red. Panels on the right show the direction of significant predictors. For (A), boxplots show medians (thick horizontal lines) with first and third quartiles (boxes), 95% confidence intervals (whiskers) and one outlier (point); asterisks indicate mean values of each group. For (B) and (C), a linear relationship is shown based on model-averaged coefficients. Household wealth is derived from a Material Style of Life principal components axis described in Figure S2.

General linear models identified fishing livelihoods and household wealth as the strongest drivers of household food security (Figure 1). Fishing households consumed more animal protein (5.3 days week^−1^) than non-fishing households (3.5 days week^1^, Figure 1a) and wealthier households consumed more diverse diets (Figure 1b) and displayed less coping behaviours than poorer households (Figure 1c). The proximity of a marine reserve did not influence any measure of food security in the multi-model information-theoretic approach (Figure 1).

**Figure 2:**
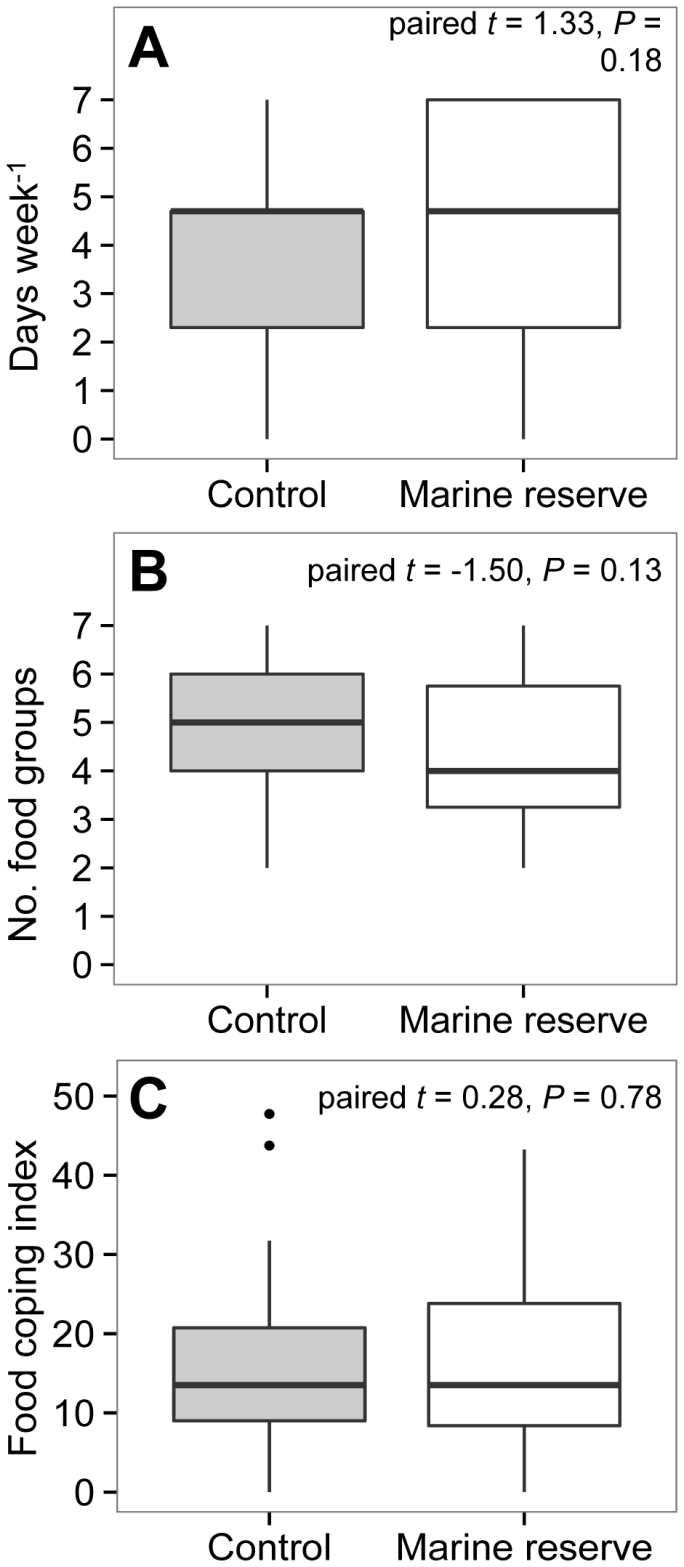
Comparisons of (A) protein consumption, B) diet diversity and C) food coping strategies between households near and far from marine reserves in Kenya (n = 54 pairs of households matched on household size, number of jobs, fortnightly expenditures and wealth). Paired *t*-tests are given for each comparison.

Matching methods identified 54 balanced pairs of treatment (near) and control (far from marine reserve) households with comparable household size, number of occupations, wealth and fortnightly expenditures (Table 3). Within this matched sample (n = 54 pairs, 108 households), there were no differences in protein consumption (paired *t*-test, *t* = 1.28, *p* = 0.20, Figure 2a), dietary diversity (*t* = −0.66, *p* = 0.51, Figure 2b) or food coping strategies (*t* = −0.21, *p* = 0.83, Figure 2c) between households near and far from marine reserves (Figure 2).

**Table 3:** Comparison of socio-economic characteristics of Kenyan coastal households near and far from marine reserves, before and after matching.

Characteristic	Sample	Mean treatment (marine reserve)	Mean control	Difference in mean values	Mean eQQ difference^a^	Mean eCDF difference^b^	*t*	*t*-test, *p*-value	KS^c^	KS, *p*-value
Household size, people	After matching	7.35	7.17	0.19	0.70	0.02	0.55	0.59	0.06	0.99
	Before matching	7.35	6.31	1.05	1.20	0.05	1.43	0.16	0.15	0.27
No. of jobs	After matching	2.67	2.46	0.20	0.20	0.03	0.95	0.34	0.11	0.37
	Before matching	2.67	2.14	0.53	0.57	0.08	2.73	**0.01**	0.21	**0.02**
Wealth	After matching	−0.97	−0.86	−0.10	0.23	0.04	−0.71	0.48	0.09	0.89
	Before matching	−0.97	0.88	−1.85	1.78	0.25	−4.94	**<0.001**	0.46	**<0.001**
Fortnightly expenditures, KES	After matching	4739.19	4633.85	105.33	321.37	0.03	0.97	0.34	0.13	0.72
	Before matching	4739.19	4597.40	141.79	281.48	0.02	0.28	0.78	0.08	0.97

a Mean difference in the empirical Q-Q plot of treatment and control groups.

b Mean difference in the empirical Cumulative Distribution Functions of treatment and control groups.

c Non-parametric Kolmogorov-Smirnov test between treament and control groups.

Statistical matching (see Methods) identified pairs of similar households and reduced differences in key characteristics between households near and far from marine reserves to allow a relevant comparison of food security metrics using paired *t*-tests (Figure 2).

**Figure 3:**
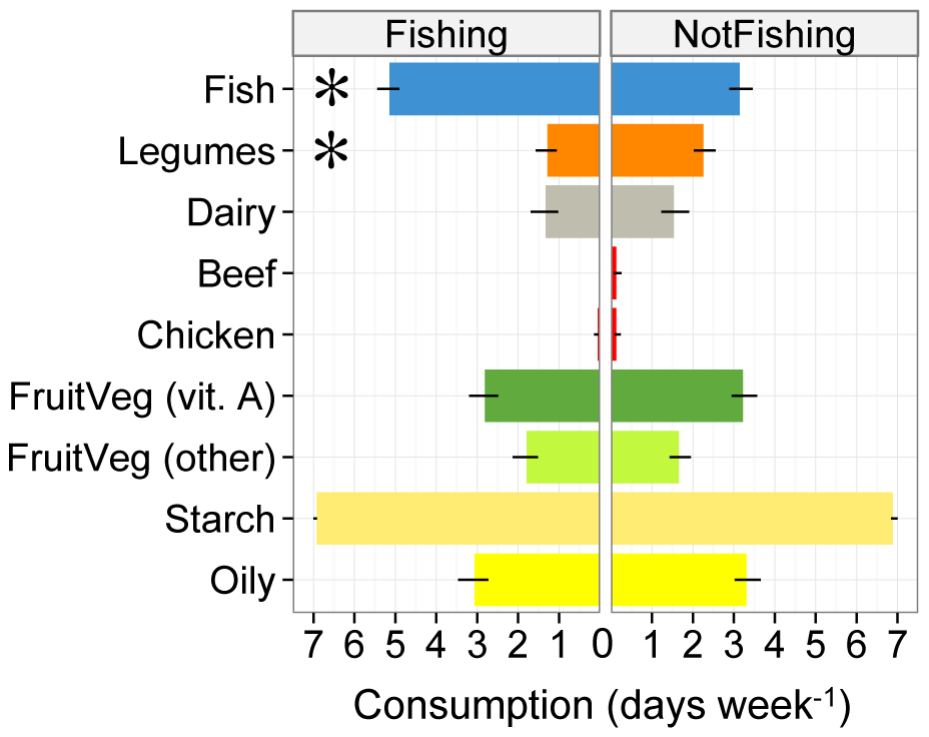
Diets of Kenyan coastal households with and without fishing as the primary livelihood. Diets are described in terms of weekly consumption (number of days consumed per week) of seven major food groups. Asterisks indicate significant differences in post-hoc comparisons after controlling for multiple tests (n = 53 fishing households, n = 58 non-fishing households).

Across all 113 households, fishing households consumed significantly different diets than non-fishing households (MANOVA, *F* = 6.25, *df* = 1,109, *p* = 0.001; Figure 3). Post-hoc tests corrected for multiple comparisons revealed that fishing households consumed more fish (*W* = 2313.5, P<0.001) while non-fishing households consumed more beans (*W* = 1151.5, P = 0.03); weekly consumption of all other food groups was similar. There were no differences in diet between households near and far from marine reserves (MANOVA, *F* = 1.10, *df* = 1,109, *p* = 0.37) or any indication that fishing households consumed different diets near a marine reserve (MANOVA fishing×reserve interaction, *F* = 1.73, *df* = 1,109, *p* = 0.15).

## Discussion

Understanding the social and economic success of management actions for local communities is a critical question for conservation [11], [55]. In this study, I found no evidence that two old, well-enforced, no-take marine reserves influenced household food security in coastal fishing communities in Kenya. Instead, food security (measured by protein consumption, diet diversity and food coping strategies) was higher in households where fishing was the primary livelihood and wealthier households.

The lack of effect for marine reserves on household food security could be explained if the marine reserves adjacent to the focal villages are not ecologically or economically effective. This does not appear to be the case. The Malindi and Kisite Marine National Parks are some of the oldest and most effective in Kenya with productive coral reef fish communities that contain more fish biomass (1643 kg/ha and 711 kg/ha biomass, respectively; [6]). In the Mombasa Marine National Park (also in Kenya), higher fish biomass inside the reserve provided more stable and valuable yields that increased revenue and income for fishermen [20], [33]. Marine reserves may also provide benefits to local communities through increased tourism. A study from the late 1990s found that households adjacent to the Kisite MNP had higher food security associated with greater tourism employment [31]. Interestingly, I did not see an effect of tourism in this study over a decade later; in this study, only four households (out of 113, or 3.5%) identified tourism as an important occupation, which may reflect declining tourism in Kenya by 2010 associated with political instability. Thus, while there is evidence that marine reserves in Kenya can have ecological and economic benefits for fisheries and tourism, I did not find evidence that these benefits have ‘spilled over’ into local communities to increase household food security.

However, while marine reserves did not increase food security, they also did not decrease food security, particularly in more remote and, on average, poorer households near marine reserves (Table 2). Marine reserves have costs for local communities following the loss and displacement of fishing grounds and imposed limits on resource exploitation that can create or reinforce poverty traps and increase food insecurity [24]. I did not find higher food insecurity near marine reserves, suggesting that the ecological and economic benefits of Kenya's marine reserves may buffer households from social costs. Studies from the Solomon Islands [26], the Philippines [21] and Tanzania [23] also found that marine reserves did not have a negative impact on human health and welfare. The current study differs from these in our focus on household food security. Overall, this may be positive news for conservation efforts: after several decades of protection, there can be ecological and economic benefits of no-take marine reserves that do not result in social costs. However, whether neutral social outcomes are an effective measure of conservation success depends on the explicit definitions of success and failure, which are often based on exclusively biological impacts and not social outcomes [25], [55].

The social context of marine reserves in Kenya might also explain the limited effects of marine reserves on food security. Households near marine reserves were poorer and relied on more occupations in both fishing and non-fishing households compared to households further from marine reserves (Figure 2). However, communities near the Kisite and Watamu marine reserves are also located in more remote areas >80 km away from the nearest city, Mombasa, which is a common challenge for impact evaluation of protected areas in developing countries [50]. For example, remote communities are further from primary markets with higher costs to transport and maintain fresh catch as compared to communities that are closer to markets [6]. Similar to other socioeconomic studies of Kenya's coral reef fishery, we used non-fishing households as a control for the effects of urbanization and distance to market [38]; our results reveal an effect of urbanization on household wealth and occupational diversity. While urbanization is an important driver of social-economic context along Kenya's coast and requires further investigation, statistical approaches to matching on household wealth and number of occupations provided appropriate household controls to disentangle a true comparison of food security near and far from marine reserves (Figure 2; Table 3).

Gender dynamics may have affected the ‘socio-economic spillover’ of fishery benefits from marine reserves to household food security. In Kenya, men largely control the coral reef fishery and the income it generates, while women typically control a household's finances and make decisions about food security. A sharply gendered division of labour in coastal and marine livelihoods is common in small-scale fisheries and can be a result of contemporary changes in the political economy of resource use. For example, in rural littoral Eastern Indonesia during the 20^th^ century, increased fishing effort and social transformations led to fisheries becoming increasingly male-oriented, leading women to often stay home to keep their children in school, take care of the household and prepare meals [56]. When men control fishing and livelihood incomes there can be limits as to whether this income actually reaches the household to benefit food security. For example, Geheb et al. [57] investigated small-scale fishing communities on Lake Victoria in western Kenya and found that male-generated fishing income generally did not reach the female heads-of-household; instead, male fishers spent this income on alcohol, cigarettes and prostitutes. This is typical of other development studies that find income can be spent differently by men and women, where women are more likely to spend money on food, healthcare and education as compared to men [58]. Thus while fishermen may be benefitting from increased income and catch near marine reserves, these benefits may not always make it to the household to increase food security as predicted. However, women can and still do play an important and often distinct role in small-scale fisheries, such as gleaning intertidal invertebrates, fishing part time, or buying and cooking fish to sell – e.g., female fish traders in Kenya are called *mama karangas*, translated to ‘frying women’ [59]. Globally, women's participation in small-scale fisheries may be largely unrecognized yet especially vital for family food security [60], [61]. Future studies are needed to trace the fine-scale movement of income between male earners and female providers and how this can food security, as well as investigating the contributions of women to coral reef fisheries and food security (e.g., [61]).

While marine reserves did not influence household food security, fishing livelihoods and household wealth did affect food security. Fishing families consumed more protein than non-fishing families, and wealthier households had greater diet diversity and relied on fewer coping strategies than poorer households. Fishing can support household food security by both providing a daily catch of essential proteins, fatty acids, vitamins and micronutrients, as well as a daily cash income that can be used to purchase other food items [62]. Subsequently, fishing households may struggle less to meet daily protein requirements than non-fishing households. For example, marine fish can provide more than twice as much protein per 100 g than legumes, the common protein consumed by non-fishing households in this study (Figure 3; 20.8 g average protein in seven species of marine fish per 100 g versus 8.7 g per 100 g of kidney beans, [63]). Household wealth also increased food security – wealthier households had more diverse diets and coped less with food insecurity than poorer households. Wealthier families likely have more income and purchasing power to buy food items and avoid food shortages compared to poorer households [64], [65]. In developing countries like Kenya, household wealth can also indicate social status [66], [67], which has also been shown to be a good predictor of household food security in southern Africa [64].

Overall, the interplay between livelihoods, wealth and poverty, urbanization and gender dynamics create a complex socio-economic landscape to understand the effects of marine conservation on food security. The social and economic context of conservation is critical to consider when attempting to elucidate the interactions and outcomes of linked socio-ecological systems, like food security from coral reef fisheries [13]. The reality of these complexities cannot be overlooked when considering the role of conservation actions for human wellbeing and food security. It is also important to consider the timeframe of these effects – for example, whether the short-term costs of establishing new marine reserves can often overcome by longer-term ecological and economic benefits, i.e. the business model for marine reserves [68].

Increasing fisheries effort and dependence on declining marine resources will increase food insecurity and conflict, especially in developing countries [69]. Finding solutions that can support subsistence livelihoods and food security in coastal communities is a critical challenge for marine resource management and conservation. No-take marine reserves are a tool that has been shown to have positive ecological and economic impacts, but these interventions may not always achieve social outcomes such as food security. Increasing gear diversity, diversifying catch portfolios of productive species, and reducing fishing effort through alternative livelihoods should also be considered when attempting to increase food security for small-scale fisheries [13], [33], [69], [70]. Sustainable development initiatives that support education, health and food security of women may also have more direct benefits to a household's welfare than leaky or indirect links from conservation and management actions [58]. Overall, complex social landscapes of urbanization, gender and power within small-scale fisheries are often overlooked yet can crucially affect how ecosystem services and social institutions support food security and human wellbeing in coastal communities [57], [61], [71].

## Supporting Information

File S1 Contains the following files: **Figure S1.** Map of study sites along the coast of Kenya, East Africa. **Figure S2.** Principal Components Analysis of household attributes to assess Material Style of Life. **Table S1.** Summary of socio-economic characteristics and food security metrics from four coastal villages in Kenya. **Table S2.** Comparison of Food Coping Strategies Index weights between published estimates for sub-Saharan Africa and this study.
(DOCX)
