# Supplementary material for: Assessing the Effect of Marine Reserves on Household Food Security in Kenyan Coral Reef Fishing Communities
Source: PLoS One. 2014 Nov 25;9(11):e113614. doi: 10.1371/journal.pone.0113614 (PMC4244085; doi:10.1371/journal.pone.0113614)
Supplement: File S1 — Contains the following files: Figure S1. Map of study sites along the coast of Kenya, East Africa. Figure S2. Principal Components Analysis of household attributes to assess Material Style of Life. Table S1. Summary of socio-economic characteristics and food security metrics from four coastal villages in Kenya. Table S2. Comparison of Food Coping Strategies Index weights between published estimates for sub-Saharan Africa and this study. (DOCX) [file pone.0113614.s001.docx]

**Supporting Information**

Figure S1. Map of study sites along the coast of Kenya, East Africa. Marine National Parks and Reserves are indicated by green outlines and text. Mombasa Marine Park and Mombasa city are shown on the map for reference, but are not study sites. Two fishing communities are located < 5km from Watamu Marine National Park and Kisite Marine National Park, respectively (near MPA sites, red: Uyombo and Anzwani). Msumarini and Kirudi villages are located > 50 km away from Watamu and Kisite reserves, and ~25 and ~20km away from the Mombasa reserve, respectively (far from MPA sites, orange).


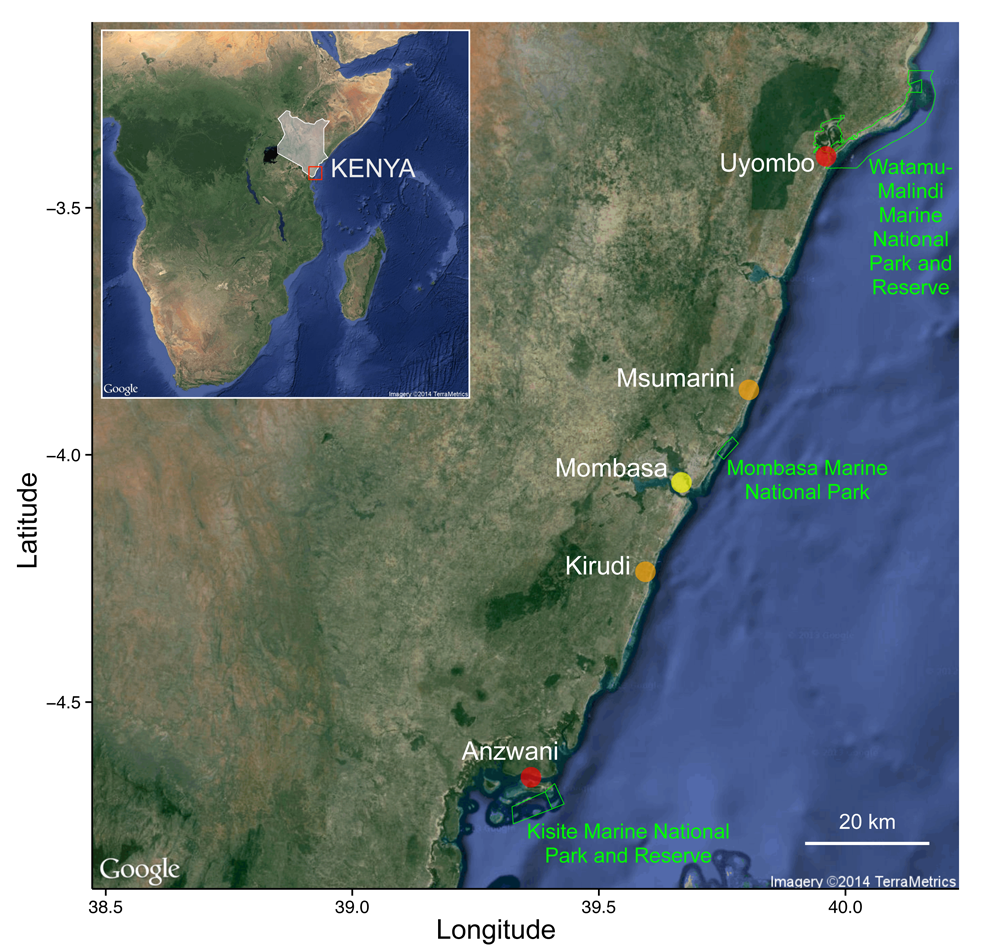


Figure S2. Principal Components Analysis (PCA) of household possessions and attributes to assess Material Style of Life for 113 rural coastal households in Kenya. The first axis, PC1, was used as a multivariate indicator for household wealth in all analyses. This axis primarily contrasts poorer households, with houses having thatch roofs, mud floors and dirt walls, and wealthier households, with houses having cement floors, cement walls and metal roofs and access to electricity and latrines.

Table S1. Summary of socio-economic characteristics and food security metrics from four coastal villages in Kenya. Mean values and standard deviations are shown. Diet diversity counts the number of seven major food groups consumed over a three-day recall (with a maximum score of 7). Food coping index assesses the number of coping strategies used by a household to deal with food shortages; higher scores indicate more coping and less food security.

| **Village** | **Marine reserve** | **Village size^a^** | **No. surveys^a^** | **Fishing, %^b^** | **House-hold size** | **No. adults** | **No. children** | **Age^c^ (years)** | **Edu. (years^d^)** | **No. of jobs** | **Wealth (PC1)** | **Fortnightly expenses (KSh)** | **Protein consumed (days week^-1^)** | **Diet diversity (no. food groups)** | **Food coping index** |
| --- | --- | --- | --- | --- | --- | --- | --- | --- | --- | --- | --- | --- | --- | --- | --- |
| Kirudi | No | ~400 | 32 | 34.38 | 6.22 (3.05) | 2.06 (0.91) | 4.22 (2.81) | 40.5 (12.2) | 2.94 (3.93) | 2.28 (1.08) | 1.46 (2.55) | 4834.09 (2616.49) | 3.8 (2.29) | 5.16 (1.19) | 15.17 (11.08) |
| Msumarini | No | ~200 | 27 | 29.63 | 6.41 (3.34) | 2.37 (1.28) | 4.07 (2.87) | 43.63 (17.78) | 1.11 (2.31) | 1.96 (0.71) | 0.20 (2.15) | 4316.85 (2557.15) | 4.07 (2.39) | 4.59 (1.28) | 16.64 (9.17) |
| Anzuwani | Kisite | 135 | 30 | 76.67 | 6.63 (2.55) | 1.9 (0.31) | 4.8 (2.52) | 41 (11.97) | 1.5 (2.53) | 2.60 (0.77) | -1.13 (1.30) | 3833.73 (1424.08) | 4.67 (2.61) | 4.23 (1.41) | 17.77 (9.3) |
| Uyombo | Watamu | 226 | 24 | 54.17 | 8.25 (5.97) | 3.12 (2.25) | 5.67 (3.92) | 32.33 (11.72) | 6.12 (3.42) | 2.75 (1.45) | -0.76 (1.64) | 5871 (3548.28) | 5.35 (1.89) | 4.71 (1.40) | 15.76 (13.21) |

^a^Number of households

^b^Percent of households with fishing as the primary occupation

^b^Age of respondent

^c^Years of education

Table S2. Comparison of published weightings of six food coping behaviours from sub-Saharan Africa with perceived severity as reported by the respondents in this study.

| **Coping behaviour** | **Maxwell 1996, 3-point scale^a^** | **Respondents' score in this study,**  **3-point scale^b^** |
| --- | --- | --- |
| Eating less preferred foods | 1 | 2.07 (0.80) |
| Limiting portion sizes | 1 | 1.95 (0.77) |
| Borrowing food or money to buy food | 2 | 2.23 (0.86) |
| Preparing food only for the children | 2 | 2.21 (0.81) |
| Skipping meals | 2 | 1.97 (0.83) |
| Going without food for whole days | 3 | 2.71 (0.55) |
| ^a^1 is least severe and 3 is most severe |  |  |
| ^b^Mean (standard deviation) of 113 responses on a 3-point scale | | |
